# CMAUP database update 2024: extended functional and association information of useful plants for biomedical research

**DOI:** 10.1093/nar/gkad921

**Published:** 2023-10-28

**Authors:** Dongyue Hou, Hanbo Lin, Yuhan Feng, Kaicheng Zhou, Xingxiu Li, Yuan Yang, Shuaiqi Wang, Xue Yang, Jiayu Wang, Hui Zhao, Xuyao Zhang, Jiajun Fan, SongLin Lu, Dan Wang, Lyuhan Zhu, Dianwen Ju, Yu Zong Chen, Xian Zeng

**Affiliations:** Department of Biological Medicines & Shanghai Engineering Research Center of Immunotherapeutics, Fudan University School of Pharmacy, Shanghai 201203, China; Department of Biological Medicines & Shanghai Engineering Research Center of Immunotherapeutics, Fudan University School of Pharmacy, Shanghai 201203, China; Department of Biological Medicines & Shanghai Engineering Research Center of Immunotherapeutics, Fudan University School of Pharmacy, Shanghai 201203, China; Department of Biological Medicines & Shanghai Engineering Research Center of Immunotherapeutics, Fudan University School of Pharmacy, Shanghai 201203, China; Department of Biological Medicines & Shanghai Engineering Research Center of Immunotherapeutics, Fudan University School of Pharmacy, Shanghai 201203, China; Department of Biological Medicines & Shanghai Engineering Research Center of Immunotherapeutics, Fudan University School of Pharmacy, Shanghai 201203, China; Department of Biological Medicines & Shanghai Engineering Research Center of Immunotherapeutics, Fudan University School of Pharmacy, Shanghai 201203, China; Department of Biological Medicines & Shanghai Engineering Research Center of Immunotherapeutics, Fudan University School of Pharmacy, Shanghai 201203, China; Department of Biological Medicines & Shanghai Engineering Research Center of Immunotherapeutics, Fudan University School of Pharmacy, Shanghai 201203, China; Department of Biological Medicines & Shanghai Engineering Research Center of Immunotherapeutics, Fudan University School of Pharmacy, Shanghai 201203, China; Department of Biological Medicines & Shanghai Engineering Research Center of Immunotherapeutics, Fudan University School of Pharmacy, Shanghai 201203, China; Department of Biological Medicines & Shanghai Engineering Research Center of Immunotherapeutics, Fudan University School of Pharmacy, Shanghai 201203, China; The State Key Laboratory of Chemical Oncogenomics, Key Laboratory of Chemical Biology, Tsinghua Shenzhen International Graduate School, Tsinghua University, Shenzhen 518055, China; Qian Xuesen Collaborative Research Center of Astrochemistry and Space Life Sciences, Institute of Drug Discovery Technology, Ningbo University, Ningbo 315211, China; Qian Xuesen Collaborative Research Center of Astrochemistry and Space Life Sciences, Institute of Drug Discovery Technology, Ningbo University, Ningbo 315211, China; Department of Biological Medicines & Shanghai Engineering Research Center of Immunotherapeutics, Fudan University School of Pharmacy, Shanghai 201203, China; The State Key Laboratory of Chemical Oncogenomics, Key Laboratory of Chemical Biology, Tsinghua Shenzhen International Graduate School, Tsinghua University, Shenzhen 518055, China; Qian Xuesen Collaborative Research Center of Astrochemistry and Space Life Sciences, Institute of Drug Discovery Technology, Ningbo University, Ningbo 315211, China; Shenzhen Bay Laboratory, Shenzhen 518000, China; Department of Biological Medicines & Shanghai Engineering Research Center of Immunotherapeutics, Fudan University School of Pharmacy, Shanghai 201203, China

## Abstract

Knowledge of the collective activities of individual plants together with the derived clinical effects and targeted disease associations is useful for plant-based biomedical research. To provide the information in complement to the established databases, we introduced a major update of CMAUP database, previously featured in NAR. This update includes (i) human transcriptomic changes overlapping with 1152 targets of 5765 individual plants, covering 74 diseases from 20 027 patient samples; (ii) clinical information for 185 individual plants in 691 clinical trials; (iii) drug development information for 4694 drug-producing plants with metabolites developed into approved or clinical trial drugs; (iv) plant and human disease associations (428 737 associations by target, 220 935 reversion of transcriptomic changes, 764 and 154121 associations by clinical trials of individual plants and plant ingredients); (v) the location of individual plants in the phylogenetic tree for navigating taxonomic neighbors, (vi) DNA barcodes of 3949 plants, (vii) predicted human oral bioavailability of plant ingredients by the established SwissADME and HobPre algorithm, (viii) 21–107% increase of CMAUP data over the previous version to cover 60 222 chemical ingredients, 7865 plants, 758 targets, 1399 diseases, 238 KEGG human pathways, 3013 gene ontologies and 1203 disease ontologies. CMAUP update version is freely accessible at https://bidd.group/CMAUP/index.html.

## Graphical Abstract

**Graphical Abstract:**
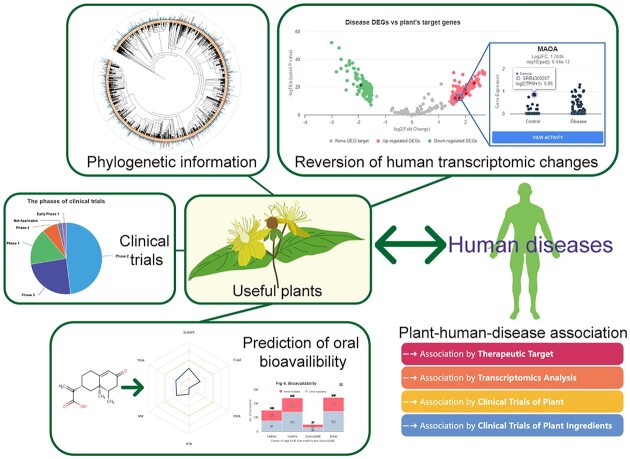

## Introduction

Functionally useful plants are vital sources of foods, medicines and other economic applications (1–3). Herbal remedies in traditional medicines, which largely employ medicinal plants, have been extensively utilized for disease curing and health maintenance (4). In recent years, well-established databases have contributed to the research of functionally useful plants especially for medical use, including HIT (5), HERB (6), TCMPG (7), SuperTCM (8), YaTCM (9), CMAUP (10), TCM-ID (11) by incorporating related data such as diseases, therapeutic uses and druggability (12).

Knowledge of the collective activities of individual plants together with the derived clinical effects and targeted disease associations is useful for plant-based biomedical research and development (13–15). Especially, an understanding of the collective activities of individual medicinal plants will be useful for functional food research, interrogations of the claimed therapeutic effects, potential mechanisms of traditional medicines (16) and discoveries of multi-component therapeutics (17). Large-scale perturbation assays of the activities of plant extracts or ingredients on individual proteins or genome-wide omics profiles provide new clues to the underlying mechanisms of functional plants (18). These efforts can be facilitated by the expanded knowledge of the collective activities of individual plants to more complex profiles such as transcriptomic changes of patients with diverse diseases (19). Moreover, analysis of differentially expressed genes (DEGs) has enabled mechanism research of complex diseases (20). Meanwhile, collective reversion of disease-related transcriptomic changes has been demonstrated to be significantly relevant to the therapeutic effects of drugs (21).

For plant-derived therapeutic ingredients, absorption, distribution, metabolism, excretion and toxicology (ADMET) properties are key indicators for their development into drug candidates (22), among which poor oral bioavailability may restrict their clinical use (23). ADMET profiles are also critical for elucidating the pharmacologically-active chemical constituents and the mode of action (MoA) of medicinal herbs (24). Therefore, predicting oral bioavailability is important for more effective drug discovery (25). Notably, many plant metabolites have undergone or are in clinical trials as anticancer agents, antioxidants and antibacterials, etc. (26–29). Evaluation of the clinical information of individual plants may provide useful insights into the comprehensive medical use of plants following the criteria of modern clinical trials. Moreover, phylogenetic information also facilitates the discovery of new botanic sources for medical use (30). Taken together, the above information can also promote the identification of plants’ potential therapeutic values and associations with diseases.

**Figure 1.**
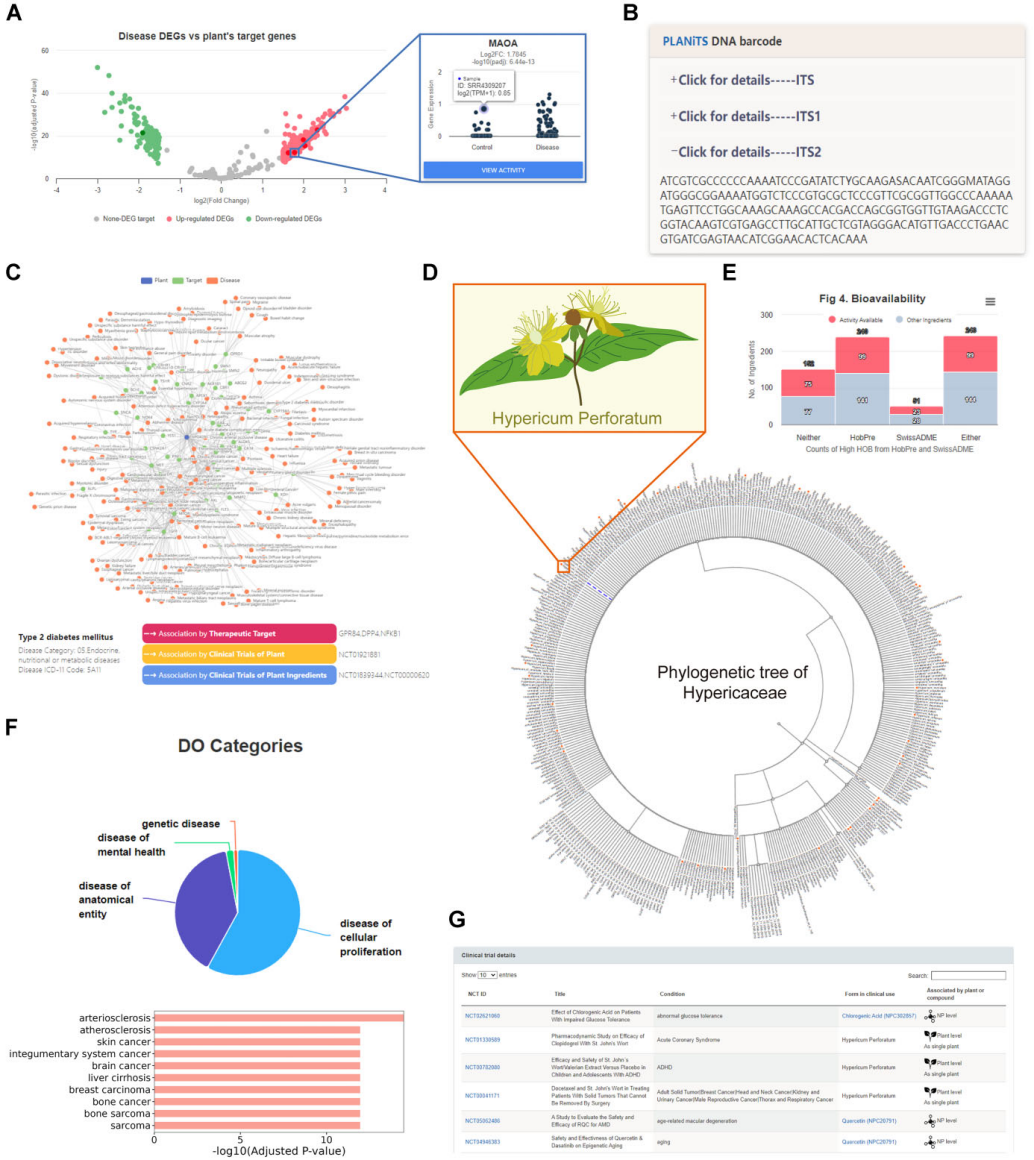
New features of CMAUP-2024 (using *Hypericum perforatum* as an example). (**A**) We evaluated the reversion of transcriptomic changes by mapping the molecular targets of *Hypericum perforatum* that overlap with DEGs of Dengue virus infection. A volcano plot was used to visualize DEGs, and for each overlapping gene, boxplots of the control and disease groups were generated for showing the distribution of log2(TPM + 1). (**B**) Internal transcribed spacer (ITS), one of the most commonly used DNA barcode markers was included in CMAUP. (**C**) The plant-human disease associations were shown in networks with four pieces of evidence: therapeutic targets, reversion of transcriptomic changes, clinical trials of plants and clinical trials of plant ingredients (only the target-based network was shown). For each associated disease, evidence was also shown (Type 2 diabetes mellitus was shown as an example). (**D**) Position and neighbors of *Hypericum perforatum* on the phylogenetic tree of *Hypericaceae* family. (**E**) Statistics of bioavailability information among individual plants. Counts of ingredients that were predicted by HobPre and SwissADME as high human oral bioavailability were shown. (**F**) Enriched Disease Ontology terms conducted by R package DOSE (https://bioconductor.org/packages/DOSE). (**G**) Clinical trial information of *Hypericum perforatum*.

Here, we updated CMAUP database by adding seven groups of new data and significantly expansion of the existing data (Table 1). The first group includes transcriptomic changes of 1152 up-/down-regulated targets of 5765 individual plants in the targeted human populations, currently covering 74 diseases based on the transcriptomic analysis of 20 027 patient samples. The second contains the information of clinical investigations of 185 individual plants in 691 clinical trials. The third covers drug development information for 4694 drug-producing plants with metabolites developed into approved or clinical trial drugs. The fourth is composed of the plant and human disease associations from four sources, including 428 737 associations by therapeutic target, 220 935 associations by reversion of transcriptomic changes in targeted human populations, 764 associations by clinical trials of individual plants and 154 121 associations by clinical trials of plant ingredients. The fifth provides the location of all individual plants in the phylogenetic tree for navigating taxonomic neighbors. The sixth contains the DNA barcodes of 3949 plants. The seventh provides the predicted human oral bioavailability of plant ingredients by the established SwissADME and HobPre algorithm. Additionally, there are 21%-107% increase of CMAUP data over the previous version (10), including 60222 chemical ingredients (2979 with potent activities <1 μM), 7865 plants, 758 targets, 1399 diseases, 238 KEGG human pathways, 3013 gene ontologies and 1203 disease ontologies. Updated new features were summarized in Figure 1. Key features of the other established databases were listed in Table 2 in comparison with this version of CMAUP.

**Table 1.** Accumulation of new features and extended data of individual plants in the latest and the previous version of CMAUP database

		CMAUP-2019	CMAUP-2024
Transcriptomic profiles		-	20027 patient samples covering 74 diseases (new)
Clinical Trials	Plant level	-	691(new)
	Ingredient level	-	14 516 (new)
DNA barcode		-	3949 (new)
Human diseases		656	1399 (increased by 113.3%)
Total plant		5654	7865 (increased by 39.1%)
Plant functional class		5 categories:	6 categories:
		- Medicinal	- Medicinal
		- Food	- Food
		- Human edible	- Human edible
		- Agricultural	- Agricultural
		- Garden	- Garden
			- Drug-producing (new)
Total ingredients		47 645	60 222 (increased by 26.4%)
Targets (activity value < 1 μM)		436	758 targets (increased by 73.9%)
System Biology	Target KEGG pathway	234 pathways	238 pathways (increased by 1.7%)
	Target Gene Ontology	2473 GO terms	3013 GO terms (increased by 21.8%)
	Target Disease Ontology	-	1203 DO terms (new)
Plant-human-disease associations	By therapeutic targets	263 130	428 737 (increased by 62.9%)
	By reversion of disease transcriptomic changes	-	220 935
	By clinical trials of individual plant	-	764
	By clinical trials of plants’ ingredients	-	154 121

**Table 2.** Different features of plant databases

Name of databases		CMAUP	TCMID	SuperTCM	IMPPAT	HERB
No. of plants		7865	10 846	6516	4010	7263
Plant functional class		Medicinal, food, human edible, agricultural, garden and drug-producing	Medicinal	Medicinal	Medicinal	Medicinal
No. of ingredients		60 222	43 413	55 772	17 967	28 212
No. of targets		758 (from experimental activity value < 1 μM)	17 512 (from HIT, STITCH, OMIM, DrugBank, text-mining)	543 (from CHEMBL)	8128 (from STITCH)	12 933 (from SymMap, HIT, TCMSP and TCMID)
No. of samples for high-throughput transcriptomic analysis		20 027	n.a.	n.a.	n.a.	6164
System biology	No. of GO terms	3013	n.a.	n.a.	n.a.	Y
	No. of KEGG pathways	238	n.a.	254	n.a.	Y
	No. of DO terms	1203	n.a.	n.a.	n.a.	8
No. of diseases		1399	2679	8634	1095	28 212
DNA barcode		Y	N	N	N	N
Phylogenetic information		Y	N	N	N	N
Latest version		2.0 (2024)	2.0 (2018)	1.0 (2021)	2.0 (2023)	1.0 (2021)

*Note: n.a. means not available; Y means this data category was included; N means this data category was not included. GO: gene ontology; DO: disease ontology.

## Transcriptomic profiles of plant targeted human diseases

In the last version of CMAUP, the collective activities of plants are primarily based on literature-reported *in-vitro* investigations. Their relevance to the regulation of human diseases needs to be evaluated with respect to such information as the human transcriptomic profiles of the plant targeted diseases (31). Indeed, it has been proved that transcriptomic changes are relevant to disease mechanisms (32), disease subtyping (33) and drug responses (34). Importantly, the reversion of disease-related transcriptomic changes was significantly associated with therapeutic effects of targeted drugs (21). This philosophy might be used to infer the collective molecular activities of plants in patient groups across various diseases. Thus, the reversion effects of plants’ ingredients on disease-related transcriptomic changes were analyzed in the updated CMAUP, by integrated analysis of >20 000 human patient samples from 74 cancerous and non-cancerous diseases.

RNA-seq is one of the most commonly used next-generation sequencing technologies for evaluating transcriptomic profiles of biological samples. In particular, these techniques are useful for probing transcriptomic changes of disease samples over control samples, which can be used to infer up- or down- regulated disease genes and therapeutic targets. With adequate RNA-seq data emerging rapidly, several uniformly processed RNA-seq data repositories have been published to remove the barriers of different deposit formats and preprocessing pipelines, thus providing resources to conduct large-scale RNA-seq meta-analysis (35–37). Therefore, combined with our collective activities of plant ingredients, the multi-target effects of individual plants can be further explored together with transcriptional characteristics of certain diseases.

**Figure 2.**
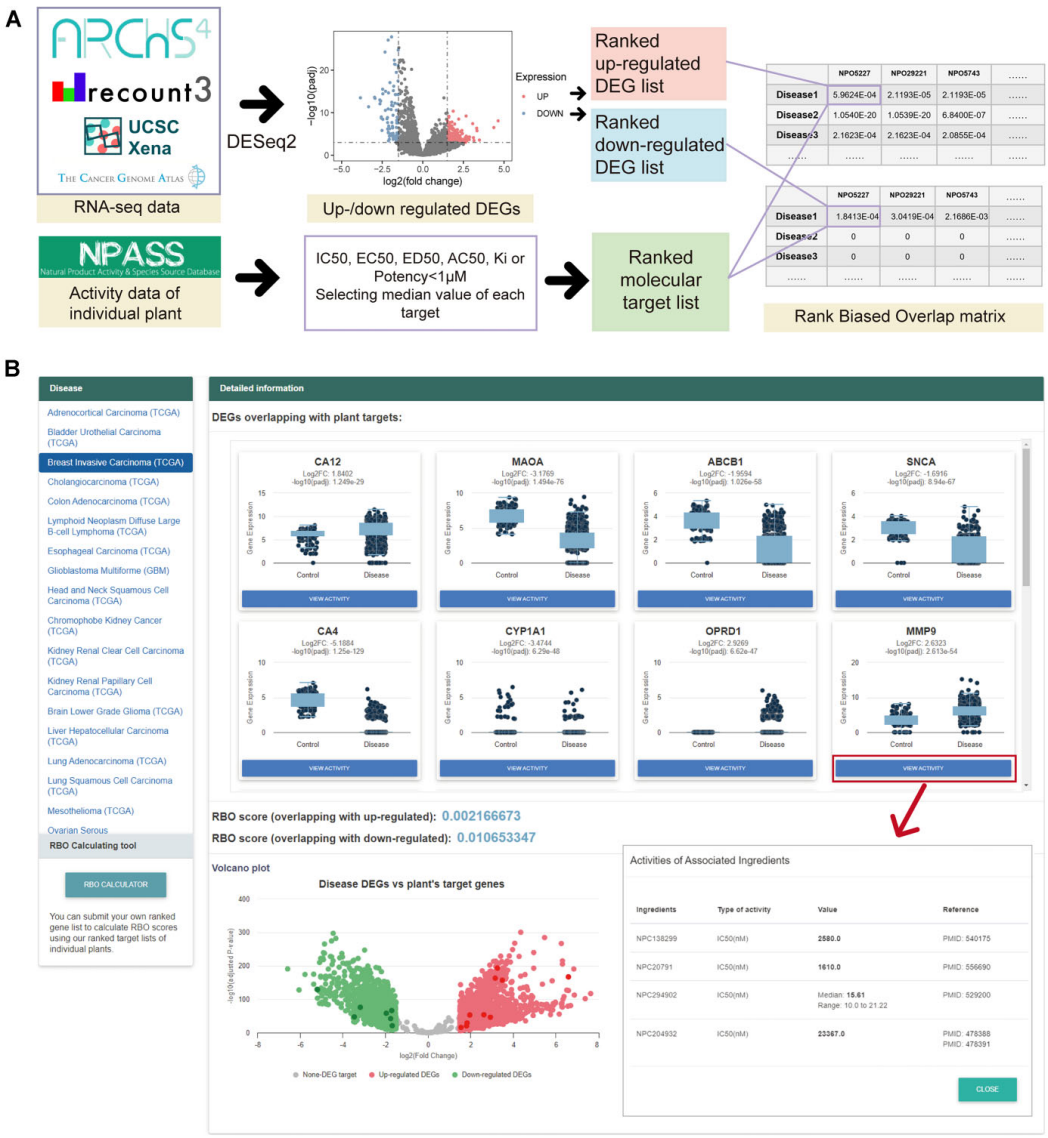
Overview of producing and visualizing reversion of transcriptomic changes of individual plants. (**A**) The pipeline of measuring reversion of transcriptomic changes. RNA-seq data were selected from ARCHS4 (36), Xena (37) and recount3 (38), among which the pre-processed data from TCGA (39) were also included. Then DESeq2 (43) was used to estimate fold change then lists of DEGs were generated (absolute value of log_2_ fold-change ≥1.5 and adjusted *P*-value < 0.001) and ranked the up-/down-regulated DEGs respectively by the absolute value of log2 fold-change from high to low. Meanwhile, the ranked list was constructed in reverse order including the molecular target list of plants (targets with IC50, EC50, ED50, AC50, Ki or Potency <1 μM and only the median value was reserved from one plant's each target for ranking). Finally, two matrixes of up-/down-regulated DEGs were generated respectively. The matrix of up-regulated DEGs can be further used to evaluate the reversion effect of transcriptomic changes. (**B**) Visualization of the patient transcriptomic changes information of 74 diseases on our website. Detailed information on DEGs was shown using boxplots. And the overview of plant targets overlapping with DEGs was shown in the volcano plot. An online RBO calculating tool was also provided for users to generate RBO scores depending on their own gene list.

In this study, a pipeline (Figure 2A) was established to identify individual plants’ potential reversion effect of disease transcriptomic changes. Firstly, patient-derived RNA-seq data from ARCHS4 (36), Xena (37) and recount3 (38), were collected, covering 74 diseases and 20 027 samples in total, which also included samples from The Cancer Genome Atlas (TCGA) (39). In total, there are 14 423 samples from the RNA-seq projects of cancers and 5604 samples from non-cancer projects. For those without adequate control samples, additional samples from UCSC Toil RNA-seq Recompute (40) were collected for consistent analysis to extract tumor samples from TCGA and control samples from GTEx (41). Next, for batch effects adjustment, ComBat-seq (42) was utilized to process samples from different research. Then differential gene expression analysis was conducted using DESeq2 (43). Genes with the absolute value of log_2_ fold-change ≥1.5 and adjusted P-value <0.001 were identified as DEGs. As shown in Figure 2B, the DEGs overlapping with molecular targets of plants were visualized using a volcano plot and a series of boxplots.

To measure the extent of gene overlap and potential reversion effects of individual plants, Rank Biased Overlap (RBO) (44) was calculated for each plant and disease using the list of up-/down-regulated DEGs and collective targets of the plant (with activity values of IC50, EC50, ED50, AC50, Ki or Potency <1 μM, collected from NPASS database (45)). The lists of up-/down-regulated DEGs were sorted in inverted order by absolute value of log_2_ fold-change (log_2_FC) respectively. Meanwhile, the lists of targets were sorted by the median of the activity values from small to large. Then for each disease-plant pair, these two lists were used as input of RBO, finally generating RBO matrixes of diseases and plants. Most of the activities of ingredients in CMAUP are inhibitory, therefore, the RBO matrix produced from up-regulated DEGs would be useful for evaluating the potential reversion effect of changes in the transcriptome. A total of 1152 overlapping target genes were identified for further visualization of expressions in disease and control sample groups. Meanwhile, an online RBO calculating tool was also constructed for users to evaluate the overlap of genes between the wanted gene list and our collective targets of individual plants.

## Clinical investigation information for plant-based medicines and products

Plant-based medicines and products have been extensively used by the populations (46). Historically, plants have contributed to a diverse pharmacologically-active chemical space and privileged scaffolds for drug discovery (47,48). In the future, plants will be still one of leading sources for drug discovery (49). In recent years, many medicinal plants and plant extracts have been evaluated in various clinical trials, providing solid evidence of connections between plants’ collective molecular activities to human diseases. For instance, it has been proved by several randomized trials that the extracts of *Hypericum perforatum* (St John's wort) have an antidepression effect (50). At the same time, a large number of individual ingredients isolated from plants and their derivatives have been tested in clinical trials. For example, Rutin, a flavonoid produced by many plants, has been clinically evaluated for its diverse pharmacological effects such as antioxidation (51).

Therefore, in order to provide a new layer of collective molecular activities of plants, the clinical trial records of plants, plant extracts and plant ingredients were incorporated into the updated CMAUP. To this end, 691 clinical trials were extracted from the ClinicalTrials.gov database (52), covering 175 individual plants and 334 diseases. In these clinical trials, whole plants or plant extracts were usually administered as the interventions, so this part of data represented the collective molecular activities of plants on patient cohorts. From the level of individual ingredients, clinical trial data were collected from ChEMBL database (53). In total, 14 516 clinical trial records of 381 plant ingredients were extracted from ChEMBL (version 32). Both the plant/plant-extract level and the individual ingredients level of clinical trial data will be quite useful for understanding the collective molecular activities of plants on human diseases and health, and thus benefit plant-based medicines and modern drug discovery. In the CMAUP, clinical trials from these two levels were collected for individual plants (if applicable) and summarized according to trial phases and ICD-11 classification of the indications (54). In addition, the comprehensive clinical trial annotations allowed us to label those plants that have been evaluated in clinical trials as the ‘drug-producing plant’, which might be very informative to guide new drug discovery from the perspectives of pharmacophylogeny and pharmacophylogenomics (55). In total, 4649 individual plants that annotated with clinical trial information were labeled as drug-producing plants.

## Human oral bioavailability prediction of plant ingredients

Plants are important sources of drugs (56), which largely attributed to the accumulated medicinal uses of abundant plants during thousands of years across the world, privileged drug-like scaffolds and the diverse pharmacologically-active chemical space (47,48). Among these key factors, the ADMET properties of individual ingredients are critical for their *in vivo* biological activities, and thus are important for plant-based medicines research and drug discovery efforts (57). For individual plants containing various ingredients, the actual amounts of components entering circulation influence the collective pharmacological effects whether they are orally administrated as a single herb or multi-herbal formula (58). Although the oral form is most commonly used for drug administration (59), many oral drug candidates failed due to poor oral bioavailability. To avoid failure in later experimental stages, validation of oral bioavailability can also be expensive and time-consuming (25). Therefore, human oral bioavailability *in silico* prediction of ingredients from individual plants can provide potential evidence for plant-derived drug discovery and the elucidation of MoA of medicinal herbs.

In the updated CMAUP, well-established algorithms were used to predict human oral bioavailability and more diverse ADMET properties. The SwissADME (60) was used for generating Bioavailability Radar from the perspectives of lipophilicity, size, polarity, solubility, saturation and saturation properties it predicted. In addition, a machine-learning-based method called HobPre was employed (58), which uses random forest models to classify molecules into groups of low oral bioavailability and high oral bioavailability. The remining ADMET properties were calculated using ADMETlab 2.0 software (61). The comprehensive annotations of ADMET properties of plants’ ingredients may facilitate to define the bioavailable and bioactive chemo-fundamentals of medicinal plants.

## Taxonomic and phylogenetic information of plants

Plant-derived drugs mostly tend to be derived from certain drug-productive families that are clustered in the phylogenetic tree (62). Herbs in traditional medicines are also phylogenetically clustered (63,64). Such clustering phenomenon is proposed as the pharmacophylogeny, which means that plants from the same phylogenetic clusters may exhibit similar pharmacological functions (55). Therefore, for those plant species or genera near drug-producing clusters or herb clusters in the phylogenetic tree, taxonomic and evolutionary evidence can be used for the discovery of new drug-producing plants or the investigation of plants with medicinal values (65). In addition to chemical constituents and taxonomic profiles, DNA markers are also important for medicinal plant phylogeny investigations (55). Various methods in DNA barcoding of plants have emerged such as single-locus barcode, multi-locus barcode and whole-plastid-based barcode (66). Collectively, providing information on plants from the taxonomy and phylogenetics perspectives may help to expand the drug-producing and medicinally-valued sources (55,64).

**Figure 3.**
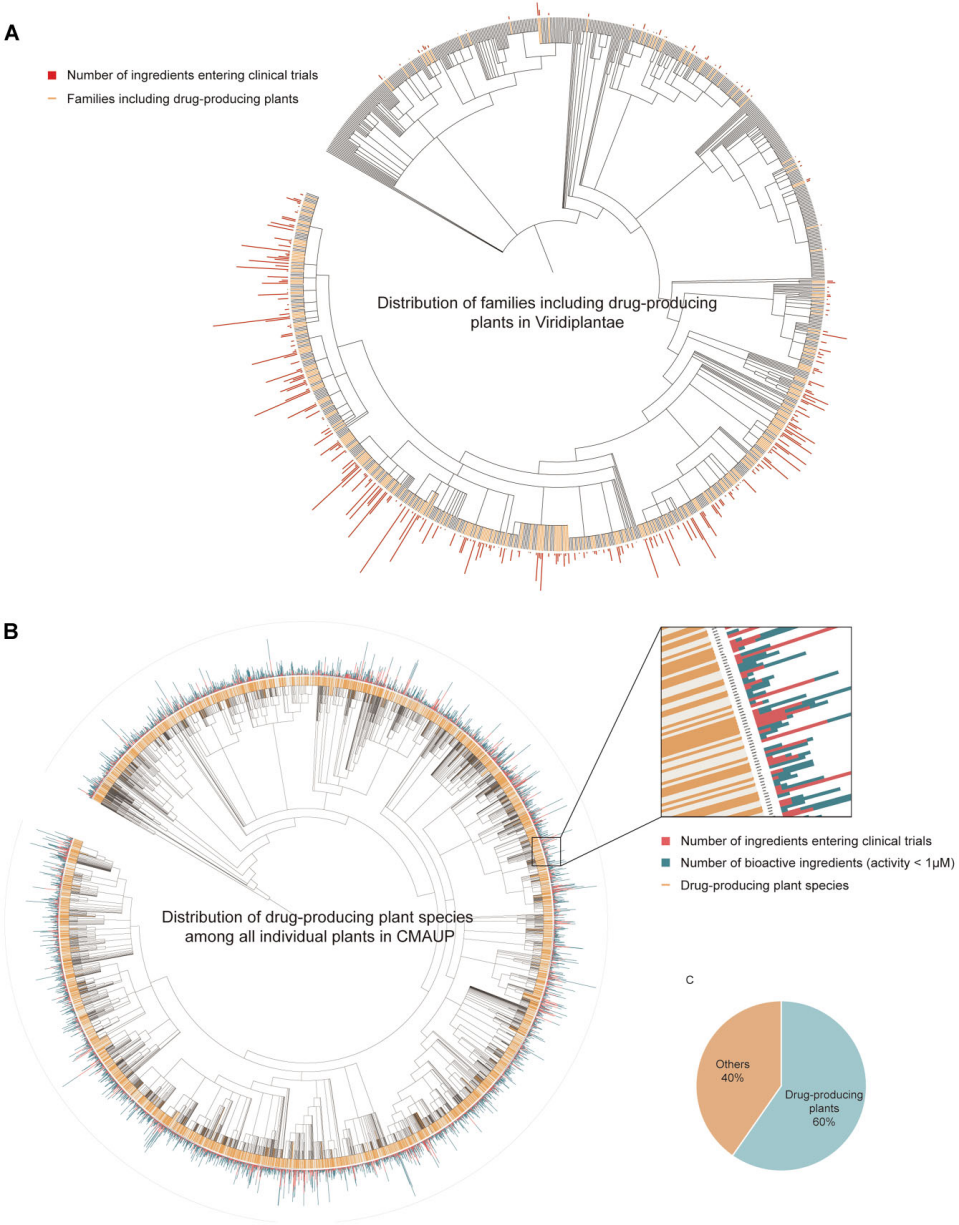
An overview of drug-producing plant distribution. PhyloT-v2 (https://phylot.biobyte.de/) and iTOL were used to generate phylogenetic trees based on NCBI taxonomic identifiers. (**A**) An overview of families that include drug-producing plant species on a family-level phylogenetic tree. A total number of ingredients entering clinical trials from each family was labeled. (**B**) An overview of drug-producing plant species on a phylogenetic tree covering all individual plants in CMAUP. The number of ingredients entering clinical trials and the number of bioactive ingredients (activity <1 μM) were labeled. (**C**) The pie chart showing the percentage of drug-producing plants and other plants in CMAUP.

Therefore, for each individual plant in the CMAUP update, we labeled functional neighbor plants within the taxonomic family. Phylogenetic tree projection of individual plants at the family level allows the overview of the distribution of bioactive plants or drug-producing plants in the pharmacophylogeny space. As shown in Figure 3, we mapped families including drug-producing plants in Viridiplantae (Figure 3A) and drug-producing plant species among all individual plants in CMAUP (Figure 3B) via phyloT (https://phylot.biobyte.de/) and iTOL web server (67). In details, we also generated phylogenetic trees for 372 families based on NCBI taxonomy (68) and labeled other relevant plant species or genera in CMAUP. In total, 60% of the individual plants in CMAUP were defined as drug-producing plants and labeled on phylogenetic trees (Figure 3C).

Meanwhile, internal transcribed spacer (ITS), one of the most widely used barcode marker, was included in the updated CMAUP. Sequences of ITS were retrieved from PLANiTS (69) database which contains Viridiplantae ITS1, ITS2 and entire ITS sequences. In total, 3949 plants in CMAUP matched corresponding ITS sequences successfully.

## Plant–human disease associations network for plant targeted human diseases

The concept of gene regulatory networks has already been widely used in human disease research (70). The development of human disease is not only attributed to the dysregulation of a single regulatory gene but also the collective changes in certain biological pathways or networks (71). In this update, plants and human diseases were linked by several layers of evidence and these comprehensive links allowed us to establish a plant–human disease association network, which may benefit the discovery of functional plants, medicinal plants and drug-producing plants from the perspective of network analysis (72).

In the CMAUP, the plant–disease association networks were constructed based on a few layers of evidence including: (i) a plant and a human disease were connected via targets, where the targets were therapeutical targets of the disease and the targets could also be potently targeted (biological activity < 1 μM) by ingredients of the plant. This layer of evidence was labeled as ‘Association by Therapeutic Target’ in the CMAUP interface; (ii) a plant and a human disease were connected via omics-derived disease-related genes, where these genes were the overlap of disease's DEGs and active targeted genes of the plant. This layer of evidence was labeled as ‘Association by Reversion of Transcriptomic Changes’; (iii) a plant and a human disease were connected via clinical trials, where the plant or plant's ingredients were evaluated in clinical trials for the disease, and were labeled as ‘Association by Clinical Trial of Plant’ and ‘Association by Clinical Trial of Plant Ingredients’ in the web-interface, respectively.

In details, we searched for and evaluated evidence between human disease and plants from four levels: therapeutic target, reversion of disease transcriptomic changes and clinical trials of plants and plant ingredients. Compared with the last version of CMAUP, we not only updated the association between each protein target and its relevant diseases from Therapeutic Target Database (TTD) (71), which included reported therapeutic targets information for treatment of diseases, but also added other three categories of associations based on clinical trials and disease-related transcriptomic changes to provide more reliable evidence. For the association by therapeutic target, bioactive protein targets of the plant were defined in ‘Collective Molecular Targets’ section and target–disease associations collected from TTD database were subsequently used to build the associations between the plant and its targeted human diseases. For association by disease genes reversion, a plant and a specific disease would be associated when at least one plant targeted gene overlapped with the disease's DEGs. For clinical trials, the plant would be associated when over one clinical trial was matched with the plant or its ingredients in ClinicalTrials.gov (52) or ChEMBL database (53).

To visualize these association networks, we defined each individual plant as the root node and its relevant diseases as leaf nodes. To build the bridge between each individual plant and its associated diseases, we adopted two types of internal nodes to establish the networks: gene symbols for targets and ClinicalTrials.gov Identifiers for clinical trials. For evidence from TTD and our newly established transcriptomic profiles, we selected the gene symbols as internal nodes to build the networks. Meanwhile, to better elucidate the linkage of plants and human diseases in clinical investigations, the detailed information of clinical trials was listed in a table and related hyperlinks were also provided for conveniently tracing back to database sources. Through network methods to visualize associations from multiple dimensions for each individual plant, association evidence of genes or clinical trials shared by various diseases could be more easily identified, thus offering new insights for biomedical research and drug development.

## Concluding remarks

The collective activities of plants manifest not only as combinations of individual targeted activities, but also as collective network regulatory activities. With the development of network approaches (73) and the generation of multi-omics data (74), new insights into the mechanisms of diseases and networks (75), and the regulation of disease networks by drugs and plant-based medicines and products (76) will be better elucidated. These data provide the foundation for the investigation of the clinically-relevant collective activities of plants against diseases (77) and the plant-human-disease associations, thereby facilitating the exploration of potential plant-derived therapies (78–80) and plant-based products (81). These in combination with the expanded molecular target information and the collective activities of useful plants can facilitate research in medicine, food, etc. (82,83). Our updated CMAUP together with other established databases (6–9) provides information about plant-human disease associations, which offers more experimental-based and computational resources to support the research on medicinal plants (84). Various databases of useful plants will continually benefit the research and development efforts for plant-derived drug discovery, food research and herbal remedies.

## Data Availability

CMAUP update version is freely accessible at https://bidd.group/CMAUP/index.html.
